# O-Töne in Fernsehnachrichten im Spannungsfeld von Narration und Argumentation

**DOI:** 10.1007/s41244-021-00201-7

**Published:** 2021-04-13

**Authors:** Martin Luginbühl

**Affiliations:** Deutsches Seminar, Universität Basel, Basel, Schweiz

**Keywords:** Narration, Argumentation, Fernsehnachrichten, Berichterstattungsmuster, News Narratives, Journalistische Kultur, Kontrastive Textlinguistik, Narration, Argumentation, TV News, Reporting Patterns, News Narratives, Journalistic Culture, Contrastive Genre Studies

## Abstract

Der Beitrag erläutert – vor dem Hintergrund des re-konstruktiven Charakters medialer Berichterstattung (Abschnitt 1) – das Konzept der ›News Narratives‹ (2). Dann wird auf die Rolle und bisherigen Untersuchungen von O‑Tönen und deren narrative Integration eingegangen (3). Auf Erläuterungen zu Fragestellung, Korpus und Methode (4) folgen quantitative und dann ausführlicher exemplarische qualitative Analysen (5). Hier werden Beiträge der Schweizer »Tagesschau« und der amerikanischen »CBS Evening News« seit den 1960er-Jahren analysiert. Es zeigt sich, dass die Beiträge zunehmend narrativ in einem engeren Sinn gestaltet werden, dass diese Entwicklung in den USA früher stattgefunden hat und dass die argumentative Funktionalisierung von O‑Tönen rekonstruiert werden kann, aber implizit bleibt.

## Abstract (en)

Against the background of the re-constructive character of media reporting (section 1), this article explains the concept of ›news narratives‹ (2). Then the role and previous research of sound bites and their narrative integration are discussed (3). Explanations of the research question, corpus and method (4) are followed by quantitative and then more detailed exemplary qualitative analyses (5). Here, contributions from the Swiss »Tagesschau« and the American »CBS Evening News« since the 1960s are analyzed. It becomes apparent that the coverage is increasingly narrative in a narrower sense, that this development took place earlier in the USA, and that the argumentative functionalization of original sound clips can be reconstructed but remains implicit.

## Journalistische Berichterstattung – Re-Konstruktion – Narration

The world that we have to deal with politically is out of reach, out of sight, out of mind. It has to be explored, reported, and imagined. (Lippmann 1921, S. 29)

[…] the power of media lies not only (and not even primarily) in its power to declare things to be true, but in its power to provide the forms in which the declarations will appear. (Schudson 1982, S. 98)

Trotz steigender Bedeutung der Online-Berichterstattung bleiben Fernsehnachrichten eine zentrale gesellschaftliche Informationsquelle, was sich in der Coronakrise in massiv gestiegenen Einschaltquoten entsprechender Sendungen gezeigt hat.^1^ Politische Themen sind in den meisten dieser Sendungen immer noch ein zentraler Themenschwerpunkt (s. Maurer et al. 2020), was Fernsehnachrichten zu einem wichtigen Gegenstand der Politolinguistik macht, ist doch davon auszugehen, dass Fernsehnachrichten die Weltsicht ihrer Publika nachhaltig beeinflussen (»priming«, »framing effect«, s. Johnson-Cartee 2005, S. 1–41).

In der tagesaktuellen Berichterstattung sind im Journalismus tätige Personen bei der Textproduktion^2^ darauf angewiesen, auf bereits existierende Muster der Textgestaltung zurückgreifen zu können. Diese Berichterstattungsmuster – darauf verweisen auch die obigen Zitate von Lippman und Schudson – sind keine neutralen Formen; vielmehr findet durch sie und damit durch den Akt des Berichtens zwingend eine (Re‑)Konstruktion der berichteten Ereignisse statt. Denn Journalismus kann (auch wenn er das durch seine Berichterstattungsformen inszeniert) Wirklichkeit nie direkt abbilden, da von einem Ereignis bestimmte Aspekte ausgewählt, verbalisiert, vertont, in Bilder gefasst und im Text angeordnet werden müssen. Dabei folgt die Repräsentation bestimmten Mustern, also Textroutinen, die einerseits eine effiziente Textproduktion erlauben, aber die Texte auch als Exemplare einer Textsorte erkennbar machen, welche den Anspruch hat, sich auf Ereignisse in der außermedialen Realität zu beziehen. Zentral für die folgenden Ausführungen ist nun, dass diesen Berichterstattungsformen selbst auch eine Bedeutung zukommt, die über die konkreten berichteten Inhalte hinausgeht und die gerade über ihre Vorgeformtheit über mehrere Beiträge hinweg stabil Bedeutungen realisieren bzw. durch Repetition und Habitualisierung bestimmte Formen mit Bedeutung aufgeladen werden (dazu Linke 2009, S. 1133) – etwa in Bezug darauf, welche Quellen als verlässliche Informationslieferanten etabliert werden, wessen Stimmen direkt zu Wort kommen und somit autoritativ werden, welche hingegen nicht oder nur indirekt zu Wort kommen etc. Nachrichten bieten so mehr als Fakten, sie bieten ein kulturelles Wertesystem der Berichterstattung.

## Die Welt erzählen: News Narratives

Every news story should […] display the attributes of fiction, of drama. It should have structure and conflict, problem and denouement, rising action and falling action, a beginning, a middle and an end. These are not only the essentials of drama; they are the essentials of narrative. (Executive Vice President der NBC-Nachrichten Reuven Frank 1963 in einer internen Mitteilung an die Angestellten, zit. in Epstein 1974, S. 4 f.)

Aus dem bisher beschriebenen Verständnis von Nachrichten heraus ist das Konzept der ›News Narrative‹ erwachsen (vgl. Bird/Dardenne 1988, 2009; Luginbühl et al. 2004; Johnson-Cartee 2005; Neiger/Tenenboim-Weinblatt 2016). Bereits Bird/Dardenne (1988) verweisen darauf, dass wir es bei Nachrichtentexten mit »culturally constructed narratives« (Bird/Dardenne 1988, S. 67) zu tun haben, in denen letztlich die Muster des Berichtens wichtiger sind als das Informieren über einzelne Fakten und Zahlen. Seit der Jahrtausendwende hat das Interesse an Narrativität wieder zugenommen, insbesondere unter dem Stichwort ›Storytelling‹, unter dem insbesondere die deutlich dramatisierende und emotionalisierende Rekonstruktion von Ereignissen in der Berichterstattung verstanden wird (vgl. Shim 2014; Krieken 2016).

Vor dem Hintergrund, dass Fernsehnachrichten immer eine eigene Wirklichkeit konstruieren (müssen) und dass dabei gängige Muster der Berichterstattung realisiert werden, fokussiert dieses Verständnis von Fernsehnachrichtenbeiträgen als (Kurz‑)Erzählungen oder eben News Narratives darauf, dass die Berichterstattungsmuster auf Kategorien der Narration bezogen werden können, wie z. B. die Darlegung von ›Fakten‹ durch Haupterzählerin und Untererzähler, die temporelle Sequenzialisierung und Perspektivierung von Teilereignissen und damit verbunden die Etablierung kausaler Relationen, die Nutzung verschiedener Formen der Figurenrede, die Realisierung wiederkehrender Motive oder die Bewertung von Ereignissen etc. (vgl. Luginbühl et al. 2004, S. 9–38). Es werden also Standardgeschichten erzählt, wobei sowohl eher inhaltliche Aspekte (z. B. Oppositionen von Gut und Böse; inhaltliches Framing einzelner Ereignisse) wie auch eher formale Aspekte (z. B. Tempus; Stimme on oder off; Kameraeinstellung) relevant und bedeutungstragend sein können. Diese Aspekte sind in vielen Fällen nur analytisch zu trennen. Zentral dabei ist immer der Mythos der Realitätsabbildung, die Erzählungen sind in ihrer Gestaltung immer darauf angelegt, als Erzählungen der Wirklichkeit gelesen zu werden, also als Re-Konstruktionen, welche die Wahrheit sein können (und nicht Konstruktionen wie in fiktionalen Erzählungen). Diese können dann ihren konstruktiven Charakter entweder mehr oder weniger verbergen (vgl. Luginbühl 2004). Zu beobachten ist aber immer eine »rhetoric of objectivity« (Roeh 1989, S. 162), welche auch die semiotische Inszenierung von Neutralität und Faktualität umfasst. Damit werden auch Fragen der Textsortenausgestaltung zentral, insofern (gerade bei Fernsehnachrichten) mit hochgradig konventionalisierten Formen Wirklichkeit repräsentiert wird und so die linguistische Textanalyse dazu prädestiniert scheint, die (kulturelle) Bedeutung von News Narrative-Formen zu analysieren. Buozis/Creech (2018, S. 1430) schreiben dazu: »News narratives, then, can be understood as the textual artifacts that reveal these structures [of representation, M. L.] in action.« Mögliche rhetorische Strategien können distanziertes Beschreiben (bzw. eigentlich Verkünden) sein, das Zitieren von als vertrauenswürdig inszenierten Quellen, Referenzen auf Augenzeugen etc. (Bock 2016, S. 495). Dies alles sind Aspekte dessen, was Bock (2016, S. 495) als »news-writer’s authority ›toolkit‹« bezeichnet; denn letztlich geht es in der Berichterstattung immer auch darum, die Autorität der Journalist*innen als verlässliche Berichterstattende (und somit deren Vertrauenswürdigkeit als Erzählinstanzen) geltend zu machen (vgl. Zelizer 1990). Dabei spielen dann auch professionelle Normen und kulturelle Bedingungen von Journalismus eine Rolle, wobei hier mit einer grossen Heterogenität zu rechnen ist (vgl. Bouzis/Creech 2018, S. 1431; Hanitzsch 2007).

Wie bei fiktionalen Erzählungen gibt es auch bei News Narratives eine grosse Bandbreite an Mustervarianten. Dazu werden in der einschlägigen Literatur oft auch Formen des Berichtens gezählt, die man aus einer narratologischen Perspektive nicht immer als ›Erzählung‹ bezeichnen würde (etwa das Vermelden von Aktienkursen). Zwar sind viele bereits erwähnte Aspekte in allen Nachrichtenbeiträgen relevant (es muss eine erzählende bzw. berichtende Instanz geben, das Ereignis muss auf gewisse Teilaspekte reduziert erzählt bzw. berichtet werden etc.), doch schon Bird/Dardenne (1988, S. 74) unterscheiden zwischen »chronicles […] as a record that something noteworthy has happened« einerseits und »stories« andererseits. In vielen narratologischen Studien wird der Begriff der Erzählung auf solche Texte beschränkt, in denen nicht einfach Informationen vermittelt werden, sondern in denen mindestens zwei Ereignisse oder Situationen in einer zeitlichen Sequenz angeordnet und im Sinn einer Transformation aufeinander bezogen werden (vgl. Luginbühl et al. 2004, S. 13 f.; Dunn 2005, S. 145; Neiger/Tenenboim-Weinblatt 2016, S. 145). Dies ist aber oft nur in einem Teil von Fernsehnachrichtenbeiträgen gegeben. Man kann deshalb bei Fernsehnachrichten unterscheiden zwischen einem *Erzählen in einem weiteren Sinn* (Re-Konstruktion von Wirklichkeit durch eine Erzählinstanz, zwingend verbunden mit der Organisation von Ausschnitten eines Geschehens und damit kulturell geprägter Weltdarstellung) und einem *Erzählen in einem engeren Sinn* (Vorliegen einer narrativen Themenentfaltung im Sinn von ›Zustand A – Komplikation – Resolution – Zustand B‹, oft mit einer doppelten Chronologie von erzählter Zeit und Erzählzeit) (Luginbühl et al. 2004, S. 12 f.). Während das Erzählen in einem weiteren Sinn auf alle Beiträge in Fernsehnachrichten (und journalistische Berichterstattung überhaupt) bezogen werden kann, es also die entsprechende Gestaltungsart der Wirklichkeitsdarstellung charakterisiert, so trifft das Erzählen in einem engeren Sinn unterschiedlich stark ausgeprägt auf einzelne Berichte zu. Allerdings erzählen Beiträge in Fernsehnachrichten oft auch über einzelne Elemente einer Erzählung im engeren Sinne – dies ist gerade etwa der Fall, wenn das Ende offengelassen wird oder wenn weitere Veränderungen angedeutet werden (vgl. Tenenboim 2008). Zudem haben sich in Fernsehnachrichten spezifische Formen des Erzählens i.w. und i.e. Sinn entwickelt, weshalb wir auch Fernsehnachrichtensendungen von uns nicht vertrauten Sendern meist als solche auf den ersten Blick erkennen.

Grundsätzlich können dabei News Narratives auf einem Kontinuum eingeordnet werden, von eher distanziert verkündenden Beiträgen, in denen der Akt der Narration tendenziell verborgen wird, zu deutlich erkennbar ›produzierten‹ Beiträgen (etwa mit auffälligen gestalterischen Mitteln). Prototypischerweise sind dabei Berichte, die i. w. S. erzählen, eher so gestaltet, dass die Re-Konstruktion unauffällig bleibt und tendenziell der Eindruck vermittelt wird, die Ereignisse unverändert und direkt so zu zeigen, wie sie passiert sind. Berichte, die i.e. S. erzählen, haben oft eine deutlich erkennbarere Gestaltung, etwa mit auffälligeren Bildbearbeitungen, berichtenden Personen vor Ort oder mehr kommentierenden statt berichtenden Passagen.

In allen diesen Varianten geht es immer (auch) darum, über die narrativen Strukturen diskursive Autorität zu etablieren (Bock 2016, S. 493) – sei es nun durch distanziertes Berichten und damit die Inszenierung von absoluter Objektivität, oder sei es durch die Inszenierung von Nähe (Live-Berichterstattung vor Ort, Emotionalisierung etc.) und damit Vertrauenswürdigkeit durch (mind. Pseudo‑)Vor-Ort-Berichterstattung (Luginbühl 2014, S. 339–366). Damit verbunden sind – wie oben schon angetönt – immer auch Fragen nach (Darstellungs‑)Macht und Ideologie (Buozis/Creech 2018, S. 1432). Denn Beiträge in Fernsehnachrichten sind immer eine autoritative, d.h. maßgebende Wirklichkeitsdarstellung, gerade weil Fakten so angeordnet werden, dass sie sich zu einer (als Muster bereits bestehenden) News Narrative zusammenfügen. Die Analyse von News Narratives bietet somit umgekehrt einen analytischen Zugang zur Charakteristik dieser Wirklichkeitsdarstellung.

## Die Integration von narrativen Stimmen: O-Töne

In der später folgenden Analyse interessiere ich mich für die Frage, welche argumentative Rolle O‑Töne in der politischen Berichterstattung im Kontext von News Narratives spielen. O‑Töne oder englisch »sound-bites« sind »a film or tape segment, within a news story, showing someone speaking« (Hallin 1992, S. 5). Es geht hier also um Personen, die nicht zur Redaktion einer Sendung gehören, welche in einem Beitrag direkt zu Wort kommen. Das sind – ich komme darauf zurück – Politiker*innen, Behördenvertreter*innen, Expert*innen, Bürger*innen etc. und das Gesprochene ist in der Regel eine Antwort auf eine (meist nicht mit übertragene) Frage bzw. ein Teil davon oder ein Ausschnitt aus einer öffentlichen Äusserung (etwa im Rahmen einer Medienkonferenz).^3^

O‑Töne nehmen in News Narratives eine Schlüsselposition ein, weil es sich bei ihnen narratologisch um Stimmen von ›Figuren‹ der ›Geschichte‹ mit »interner Fokalisierung« (Genette 1998, S. 134) handelt und weil sich deswegen die Einbettung derselben dazu anbietet, die Ausrichtung der ›Geschichte‹ interpretativ zu rekonstruieren. Es wird sich zeigen, dass die Funktionalisierung der O‑Töne nie eindimensional ist; diese können dazu dienen, eine informationsbezogene Aussage zu objektivieren und zu legitimieren, zu bewerten, unterhaltsam zu gestalten etc. Was aber ein unumgängliches Merkmal eines O‑Tons ist, weil die Stimme eines Menschen zu hören ist, sind Persönlichkeit und der Grad an Emotionalität: O‑Töne »capture personality and reveal inner feelings. They generate emotion. They let the journalist ›show‹ rather than ›tell‹« (Montgomery 2007, S. 5).

Zu O‑Tönen gibt es eine ganze Reihe von Studien, die sich schwerpunktmässig mit amerikanischen TV News beschäftigen und innerhalb dieser Sendungen auf O‑Töne von Politiker*innen fokussieren. Hallin (vgl. Hallin 1992) kommt bereits in seiner unterdessen klassischen Studie zum Schluss, dass die in die Berichte integrierten O‑Töne immer kürzer werden. So sinkt die durchschnittliche Dauer eines O‑Tones in der Berichterstattung der Nachrichtensendungen von ABC, CBS und NBC in den Wahljahren zwischen 1968 bis 1988 von 43,1 Sek. auf 8,9 Sek. Nachfolgestudien mit aktuelleren und anders zusammengesetzten Daten (vgl. Lowry/Shidler 1995, 1998; Russomanno/Everett 1995; Lichter 2001; Bucy/Grabe 2007; Sülflow/Esser 2014) stimmen darin überein, dass die durchschnittliche Länge heutzutage nicht über 10 Sek. liegt und tendenziell seit den späten 1980er-Jahren bis Anfang des 21. Jahrhunderts auf unter 8 Sek. gefallen ist. Im Vergleich zwischen 2004 und 2008 stellen Sülflow/Esser (vgl. Sülflow/Esser 2014) einen leichten Anstieg von 7,8 auf 8,9 Sek. fest, in meinen Daten der »CBS Evening News« ist ebenfalls ein leichter Anstieg zwischen 2005 (8 Sek.) und 2013 (11,09 Sek.) zu beobachten.^4^ Der langfristige Trend ist dennoch eindeutig: Im Vergleich zu den 1960er- und 1970er-Jahren sind die O‑Töne in der (v.a. politischen) Berichterstattung massiv kürzer geworden; ein Befund, der insbesondere auf US-amerikanische Fernsehnachrichten zutrifft. Vergleicht man diese Entwicklung mit einigen europäischen Fernsehnachrichten (von öffentlichen und privaten Sendern, vgl. Sülflow/Esser 2014), so zeigen sich zwar Unterschiede zwischen den US-amerikanischen und den europäischen Sendungen wie auch zwischen Sendungen von öffentlichen bzw. privaten Sendern (Sülflow/Esser 2014, S. 412 f.), diese Unterschiede werden aber im Laufe der Zeit zunehmend kleiner. Die Unterschiede zwischen (auch national mitbedingten) Redaktionskulturen scheinen also zu schwinden.

Soweit ich sehe, reichen die Untersuchungen der europäischen Sendungen (vgl. Esser 2008; Sülflow/Esser 2014) allerdings nicht bis ins 20. Jahrhundert zurück. Ein Blick in mein Korpus der Schweizer »Tagesschau« zeigt, dass hier die Entwicklung keinen ganz eindeutigen Trend aufweist. So sind die O‑Töne in der analysierten Woche von 1968 im Schnitt 16,79 Sek. lang. Dieser Wert steigt dann auf 23,83 Sek. für die Woche von 1986, sinkt auf 7,86 Sek. (1999) und steigt auf 13,71 Sek. für die analysierte Woche von 2013.^5^ Trotz dieser Schwankungen lässt sich aber festhalten, dass sich die O‑Töne von Politiker*innen seit den 1990er-Jahren grob um eine Durchschnittslänge von 10 Sek. herumbewegen.

Die Länge bzw. vielmehr die Kürze dieser O‑Töne, die sich in der aktuellen Berichterstattung in allen untersuchten Fernsehsendungen des 21. Jahrhunderts beobachten lässt, kann nun auf journalistische Kulturen und auf News Narratives bezogen werden und dort dann auf die Frage, inwiefern O‑Töne in entsprechenden News Narratives *argumentativ* funktionalisiert werden. Ganz grundsätzlich können die im Vergleich zu früher kürzeren O‑Töne als ein Symptom einer Berichterstattung interpretiert werden, welche zunehmend journalistisch-zentriert ist: Während bei langen O‑Tönen politischen Akteuren für relativ lange Zeit das Wort (und damit grössere Teile der Wirklichkeitsdarstellung) überlassen wird, so erlauben kürzere O‑Töne eine Berichterstattung, die unabhängiger ist von den Quellen. Damit werden News Narratives auch selbstständiger, weil die O‑Töne besser aus dem ursprünglichen Kontext extrahiert und so auf die Erzählungen zugeschnitten werden können; die journalistische Berichterstattung wird damit insgesamt autonomer und in dem Sinn interventionistischer, als Interpretationen von Journalist*innen im Vergleich zur Selbstdarstellung von Politiker*innen mehr Gewicht erhalten (vgl. Hanitzsch 2007, S. 377 f.; Sülflow/Esser 2014, S. 287). Lange O‑Töne, so Baym (2004, S. 289), haben die »narrative modality […] of stenographer«, sie vermitteln den Eindruck, die Realität unvermittelt abzubilden. Kurze O‑Töne hingegen, die mit zwingend höherer Schnittfrequenz in andere Nachrichtenbilder hineinmontiert werden, verweisen schon durch diese Form auf die Konstruiertheit der Berichterstattung und somit auch darauf, dass es sich um ein »narrative device« (Baym 2004, S. 290) handelt.

In der folgenden Analyse werde ich der Frage nachgehen, wie in der amerikanischen Sendung »CBS Evening News« und der Schweizer »Tagesschau« O‑Töne in Beiträgen zu politischen Themen argumentativ funktionalisiert werden, wobei ich auf Beiträge fokussiere, die tendenziell dem ›Erzählen im engeren Sinn‹ zugerechnet werden können, also nicht einfach faktenbezogene Informationen darlegen, sondern mindestens partiell einer narrativen Themenentfaltung folgen.

## Fragestellungen, Korpus und Methode

Im Zentrum der folgenden Analyse steht die Frage nach dem Stellenwert und Aufkommen argumentativ funktionalisierter O‑Töne in politischer Berichterstattung von Fernsehnachrichten mit einer News Narrative, die eine narrative Themenentwicklung aufweist (›erzählen i.e. S.‹). Dabei verstehe ich unter einer argumentativen Funktionalisierung eine inhaltliche Gestaltung und/oder eine sequenzielle Positionierung eines O‑Tons in einem Beitrag, der nahelegt, den O‑Ton als Begründung oder mindestens als Plausibilisierung einer These zu verstehen. Zentral ist, dass der O‑Ton eine implizierte oder explizierte These zusätzlich stützt, eine These explizit macht oder aber erst die Rekonstruktion einer impliziten These ermöglicht, indem der O‑Ton ein Strukturelement einer Argumentation realisiert (vgl. Grundler 2011, S. 18–22; Kienpointner 2008, S. 708–710) – etwa, um die Toulmin’schen Konzepte zu verwenden, eine Schlussregel, eine Stützung oder ein Datum (vgl. Toulmin 2003 [1958]). Dabei kann es sich um relevante und plausible Argumente handeln, aber auch um Trugschlüsse (Kienpointner 2008, S. 713–715).

Bezüglich der Funktionalisierung von O‑Tönen sind u.a. die Fragen relevant, wie sie in eine Narration integriert werden und inwiefern ihnen argumentative Autorität zukommt. Damit ist im Kontext einer News Narrative die Frage verbunden, inwiefern O‑Tönen eine Rolle bei der Inszenierung von Glaubwürdigkeit und somit von Authentizität zukommt (vgl. Luginbühl 2004).

Der folgenden Analyse liegt ein umfangreiches Korpus aus der Schweizer »Tagesschau« und der amerikanischen »CBS Evening News« zugrunde (genauere Angaben dazu in Luginbühl 2014, S. 137–149). Erhoben wurde jeweils eine (künstliche) Woche aus den Jahren 1968, 1978, 1982, 1986, 1991, 1999 und 2005, für diese Studie ergänzt um eine Woche aus 2013 und um ausschliesslich qualitativ analysierte Beispiele aus 2020.

Methodisch wurde so vorgegangen, dass zunächst im Korpus durch eine datengeleitete Analyse eine Kategorisierung der Personengruppen erarbeitet wurde, welche in O‑Tönen direkt zu Wort kommen. Als Basis für die qualitative Analyse wurden die O‑Töne anschliessend quantitativ analysiert. Dabei wurden die O‑Töne gezählt, in Sek. gemessen und nach Personengruppen sortiert ausgewertet.

In die Analyse aufgenommen wurden 201 Filmmeldungen und Korrespondentenberichte^6^ aus der politischen Berichterstattung, in denen Politiker*innen und/oder Behördenvertreter*innen (welche die Position von Regierenden wiedergeben) in Form von O‑Tönen zu Wort kommen^7^. Diese Beiträge wurden im Hinblick auf Narration und Argumentation analysiert. Dabei gehe ich davon aus, dass News Narratives gestalthaften Charakter haben, dass also die narrative Kohärenz durch das Zusammenwirken mehrerer analytisch zu trennender Ebenen gleichzeitig etabliert wird. Im Zentrum dabei stehen Ausgestaltung und Entfaltung des Themas; Bildauswahl und -gestaltung; Sprache-Bild-Ton-Koordination; Schnittfrequenz; Inszenierung von Aktualität, Authentizität, Nähe; Perspektivierung; Kommentierung; Formen der Redewiedergabe sowie syntaktische und lexikalische Spezifika und stimmliche Aspekte (Stimmlage, Sprechgeschwindigkeit, Akzentuierungen etc.).

## Analyse

### Quantitative Beobachtungen

Da im Folgenden die qualitative Analyse im Zentrum stehen wird, soll hier kurz auf einige zentrale Befunde der quantitativen Analyse exemplarischer Fälle eingegangen werden. In der »CBS Evening News« sind die Anteile zwischen den einzelnen Personengruppen seit den späten 70 Jahren mehr oder weniger gleich verteilt und im Schnitt in allen Gruppen um die 10 Sek. lang. Wie oben bereits angetönt liegen in der Schweizer »Tagesschau« die Dinge anders. Hier ist keine kontinuierliche Entwicklung zu beobachten, sondern eher wellenförmige Bewegungen. So kommen etwa in der Woche von 1968 Behördenvertreter (ausschliesslich männliche) im Schnitt länger zu Wort als Politiker*innen (25 vs. 16,8 Sek.), 1986 hat sich das Verhältnis deutlich verändert (5 vs. 23,8 Sek.), verkehrt sich noch einmal 1991 (18 vs. 11,5 Sek.), seit 1999 liegen die Werte näher zusammen und fallen 2013 beinahe gleich aus (14 vs. 13,7 Sek.). Aus Platzgründen kann hier nun nicht auf weitere quantitative Auswertungen eingegangen werden (Signifikanz der Unterschiede, Median etc.), doch schon die jetzt referierten Zahlen weisen auf grundlegende Unterschiede zwischen den beiden Sendungen hin, aber auch auf eine (partielle) Konvergenz seit den späten 1990er-Jahren. Zudem zeigen die Veränderungen, dass den verschiedenen Akteursgruppen im Lauf der Zeit immer weniger und im Fall der »Tagesschau« deutlich unterschiedlich viel Zeit zukommt, was – wie oben erläutert – auch mit Definitionsmacht und Autorität, mit »formal construction of discursive authority« (Baym 2004, S. 283) in der Berichterstattung zu tun hat.

### 1960er-Jahre

Die Berichterstattung der »Tagesschau« der 1960er- und 1970er-Jahre ist dadurch geprägt, dass die Gestaltung der Beiträge darauf abzielt, den Eindruck einer unvermittelten Wirklichkeitsabbildung zu vermitteln (vgl. Luginbühl 2014, S. 339–365). Die Beiträge sind dementsprechend wenig narrativ in einem engeren Sinne gestaltet, auch sind kaum Argumentationen rekonstruierbar, welche die Ereignisse deutlich interpretieren oder Kausalitäten implizieren. Im Fall von O‑Tönen bedeutet dies, dass sich der Sprechertext auf die Wiedergabe von Kontextinformationen zu einem O‑Ton und eine abschliessende Bemerkung beschränkt, wie im folgenden Beispiel vom 22. August 1968^8^ (Tab. 1), der über internationale Reaktionen über den Einmarsch russischer Streitkräfte in die Tschechoslowakei berichtet (copyright aller Abbildungen SRF bzw. CBS).

**Tab. 1:** Beispiel der Schweizer »Tagesschau« vom 22. August 1968

Maschinengeschriebener Text (Manuskript)	Film und Originalton (Transkript)
[- 1:06 Reaktionen werden berichtet aus Jugoslawien, Belgrad, Paris, dazu jeweils Filmaufnahmen der Kundgebungen]	1:06
	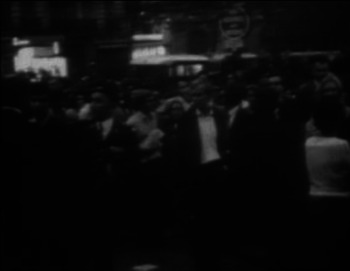
	Jemand ruft »communiste assassin«.
Kundgebung auch in Westberlin. Dort äusserte sich der regierende Bürgermeister Klaus Schütz.	
	1:13
	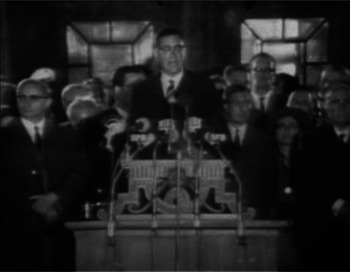
	Schütz on: Über unterschiedliche nationale Geschichten hinweg - ein friedliches Zusammenleben - in Europa für alle zu ermöglichen. DIESE Stadt lebt nicht von der Krise, diese Stadt lebt NICHT von der Gewalt, diese Stadt will Frieden. Und heute - will Berlin - den Frieden in FREIheit für das tschechoslowakische - Volk.
1:43 Soweit Klaus Schütz, der regierende Bürgermeister von Westberlin. Hier in Westberlin, so fanden in fast allen westdeutschen Städten Demonstrationen statt.	1:46
	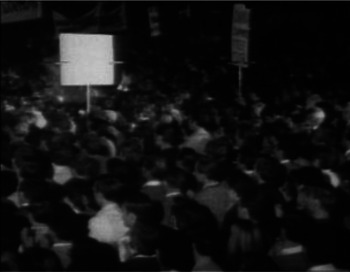
	applaudierende Menschenmenge
[…]	[…]

In diesem exemplarischen Beitrag werden die meist längeren O‑Töne kurz eingeordnet, indem berichtet wird, wer wo und in welchem Rahmen etwas gesagt hat (»Kundgebung auch in Westberlin. Dort äusserte sich der regierende Bürgermeister Klaus Schütz.«). Der Sprecher kommentiert oder paraphrasiert die O‑Töne aber nicht, sondern lässt scheinbar unbeteiligt andere Akteure sprechen.

Hier wird Objektivität und damit authentische Berichterstattung inszeniert, indem Hinweise auf die Produktion des Beitrags vermieden werden: Es dominieren distanzierte, halbnahe bis totale Kameraeinstellungen, es gibt keine auffälligen Kameraschwenks oder Bildübergänge, es wird auf auffällige rhetorische Mittel verzichtet etc. Zudem ist die Sprache-Bild-Koordination in dem Sinn eng, als die Filmaufnahmen jeweils direkt illustrierend auf den Sprechertext bezogen werden können und so das Gesagte sozusagen bezeugen und den Sprechertext zusätzlich in seinem Wahrheitsgehalt stützen. Dies gilt natürlich in besonderem Mass für den O‑Ton, als hier das Gesagte und Gezeigte zusammenfallen, der Film also die Quelle zeigt und so direkt Zeugnis abzulegen scheint von einem (Sprech‑) Ereignis. Dies tut ein O‑Ton aber nur begrenzt, nur schon, weil ja immer nur eine Auswahl zu hören ist und unklar bleibt, was sonst noch alles von wem gesagt wurde.

Bezüglich News Narrative kann festgehalten werden, dass wir hier – auch dies typisch für die Zeit – kaum O‑Töne von Politiker*innen finden in Beiträgen mit narrativer Themenentfaltung. Zudem wird keine Argumentation i.e. S. (mit rekonstruierbaren Strukturelementen wie These, Begründung, Konklusion etc.) realisiert. Vielmehr haben wir eine Berichterstattung, die eine Art direkte Transparenz auf die aussermediale Realität inszeniert und die Wirklichkeitsdarstellung autorisiert. Bock (2016, S. 439) spricht in diesem Zusammenhang von einem narrativen Stil, den sie »mimetic (showing)« nennt. Der mimetische Stil vermeidet Hinweise auf die Produktion, folgt einem (traditionellen) dokumentarischen Stil und »tend[s] to shift storytelling responsibility to their subjects« (Bock 2016, S. 497). Baym (2004, S. 284) beobachtet einen ähnlichen Stil in amerikanischen Fernsehnachrichten der frühen 1970er-Jahre und spricht von »typographic journalism«, den er so charakterisiert: »privileging the verbal, limiting the visual to narrowly mimetic representations of the real, emphasizing the voice of the lawmaker, and eliding the journalists’ own acts of mediation«.

In einem Beitrag der »CBS Evening News« vom 20. August 1968 liegen die Dinge ähnlich (Tab. 2).

**Tab. 2:** Beispiel »CBS Evening News« vom 20. August 1968

Zeit	Text (Transkript)	Film (Transkript)
0:00	Anchor on: But meanwhile the democratic platform committee ordered {?} a second day of hearings today in Washington where the Vietnam issue dominated again the proceedings. CBS news correspondent Roger Mudd reports.	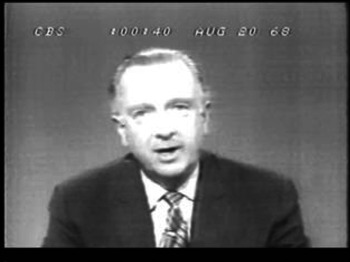
0:12	Correspondent on (im Hintergrund Rede der Versammlung zu hören, nicht verständlich): Secretary of State Dean Rusk testifies this evening at an extraordinary night session of the democratic platform committee. […] They began with senator William Fulbright of Arkansas whose appearance drew sustained applause from at least ONE third of the committee.	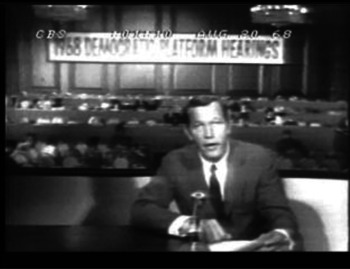
0:44 0:57	Fulbright on: As a first step for realizing these objectives – through the Paris peace negotiations, – the United States should terminate ALL bombing of Nord Vietnam and call for an immediate cease fire. Div.: Applaus	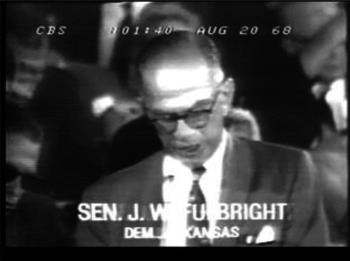
1:01	Correspondent on: But the toughest statement on the Vietnam came from the democrats NEWest presidential candidate senator George McGovern of South Dakota who has become increasingly irritated by vice-president Humphreys move to the left on the war.	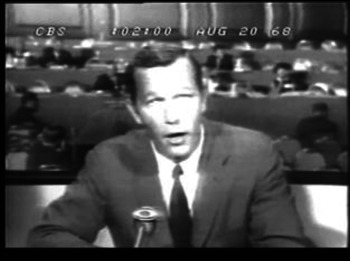
1:15	McGovern on: We call our law and order at home. – While spreading death and disorder abroad. And wasted the resources needed to lay a foundation for – law and order in our own land. We alienated our friends around the globe and divided and confused our own citizens – on an unprecedented scale. The damage to our monetary fiscal economic life is incalculable. –	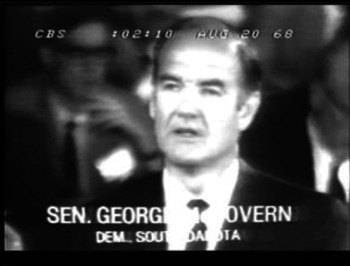
1:43	But the most compelling reason – for ending this war – is that it is a MORAL disaster for America and for the people of Vietnam.	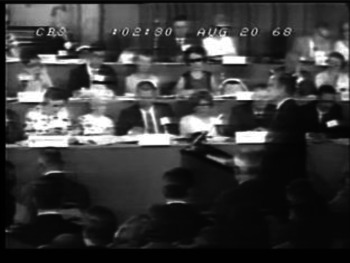
1:54	This war has become a smoldering cancer – that is eating away – at the ideals of dignity and compassion which have ALways been our surest guide from the – earliest days of the republic.	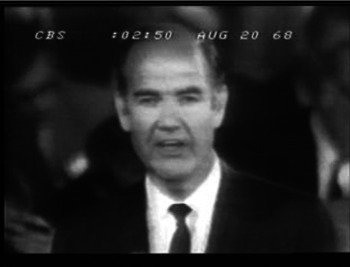
2:07	Correspondent on: There are growing indications tonight that the McCarthy, the McGovern and the former Kennedy people are now reaching an agreement on a Vietnam plank that all dissidents fractions can support. In addition, these fractions are combining to attack vice president Humphrey. A case in point was developed in this interview with former Kennedy adviser Theodore Sorensen by Dave Schoumacher.	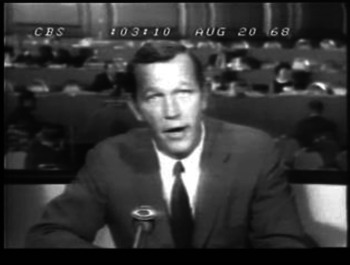
2:28	Schoumacher on: What is your view of say {?} the vice presidents positions that ehm there was no essential difference between him and senator Kennedy.	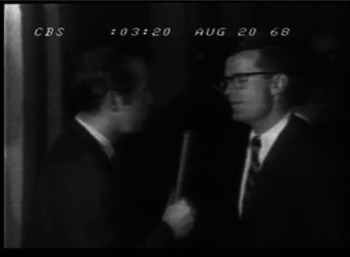
2:35	Sorensen on: I don’t wanna get into an attack on the vice president – I would simply say that – if he is/he is right, in the sense that senator Kennedy – opposed imPOSING – a coalition government of on ehm – Saigon if that indeed is a/– an/an open issue. But for the vice president to say that he therefore he’s close to Robert Kennedy’s position on Vietnam at the same time the supporting president Johnson’s – position of Vietnam is – obviously nonsense.	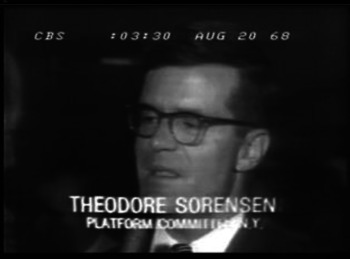
3:06 3:15	Correspondent on: Late this afternoon four other former Kennedy aides called Humphreys claim false and misleading. Roger Mudd, CBS news at the democratic platform hearings.	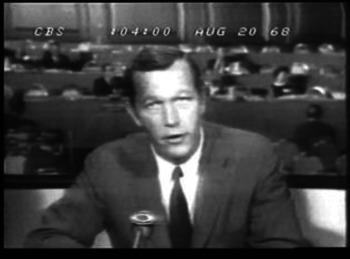

Nach der Ankündigung des Beitrags über die Parteitage der Demokraten durch den Moderator (das »But« am Anfang bezieht sich auf Proteste vor dem Veranstaltungsgebäude) informiert der Korrespondent, wer im folgenden O‑Ton in welchem Kontext der Parteitage zu hören ist. Bereits hier rahmt der Journalist den Konvent als Ort des Kampfes zwischen Anhängern und Feinden des Vietnamkrieges (»peace democrats« vs. »hard line«). Der Beitrag belegt dies dann, allerdings nicht i.e. S. argumentativ, sondern durch die Anordnung drei längerer O‑Töne, die in Bezug auf diese News Narrative in erster Linie Belegfunktion haben. Den ersten dieser O‑Töne kommentiert der Korrespondent nicht direkt, sondern beschreibt die Reaktion des Publikums (»drew sustained applause from at least ONE third of the committee«), den zweiten O‑Ton bewertet er nur sehr allgemein (»the toughest statement«), zum dritten werden wieder Reaktionen berichtet. Der Beitrag stellt die verschiedenen Positionen dar, indem er längere O‑Töne aneinanderreiht. Zwar rahmt er den berichteten Tag des Konvents, auch wird die Produktion des Beitrags mehrfach deutlich (Korrespondent im Bild, er liest von einem sichtbaren Papier ab). Dennoch ist der Beitrag in dem Sinn ›mimetisch‹ bzw. ›typographic‹, als hier wie im »Tagesschau«-Beitrag nicht die Narration im Zentrum steht (es gibt keine Koda oder einen Hinweis auf eine mögliche Entwicklung am Ende), sondern die Stimmen der Politiker und ihre Argumente, die in den O‑Tönen ausschnittweise wiedergegeben werden und so die Debatte abbilden. Im Bericht des Korrespondenten selbst wird keine Argumentation i.e.S. etabliert.

### 1990er-Jahre

Wie oben erwähnt, sind die O‑Töne von Politiker*innen in der »CBS Evening News« in den 1990er-Jahren im Durchschnitt auf unter 12 Sek. gesunken. Einher damit geht eine engere Integration dieser Stimmen in die News Narrative in dem Sinne, dass nicht mehr wie in obigem Beispiel die O‑Töne das Rückgrat der Berichterstattung ausmachen, sondern dass die O‑Töne nun passgenau für die journalistische Narration zugeschnitten und entsprechend montiert werden. Dies zeigt sich etwa in folgendem Beitrag vom 21. Januar 1991 (Tab. 3).

**Tab. 3:** Beispiel »CBS Evening News« vom 21. Januar 1991

Zeit	Text (Transkript)	Film (Transkript)
0:00 0:14	Anchor on: Official US anger and condemnation of Iraq for the mistreatment of POWs was delivered not only in public and in private, in the case of the top Iraqi diplomats still in Washington, it was also delivered PERsonally. State Department Correspondent Bill Plante has our report on that.	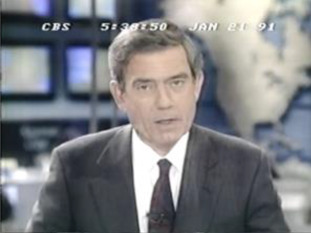
0:00	Correspondent off: Iraq’s chief diplomat in the US, summoned to the State Department for an expression of US outrage over abuse of captive Americans,	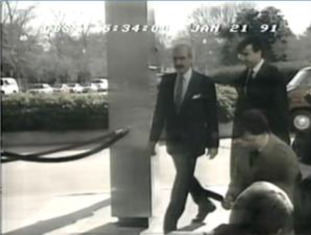
0:09	shrugged off charges that Baghdad is violating the	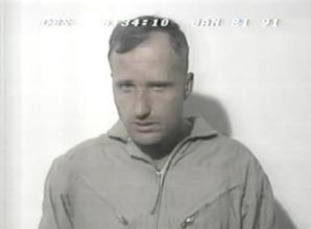
0:12	Geneva agreements on treatment of POWs.	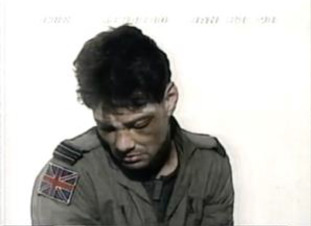
0:15	Al-Shewayish on {spricht mit Akzent}: Iraq respects all international conventions.	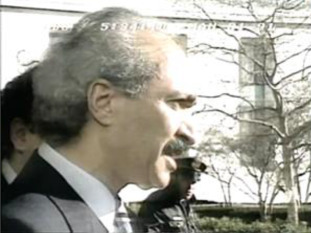
0:18	Correspondent off: But Iraq’s ambassador to Britain stripped away the language of diplomacy when	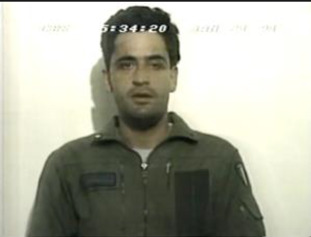
00:22	HE was asked about the POWs. Al-Salihi on {spricht mit Akzent}: If you want be safe, – avoid attacking our country.	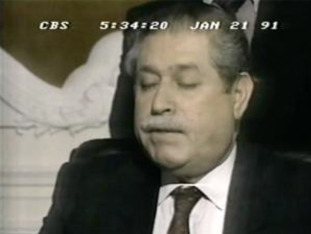
0:28	Correspondent off: There is little doubt here that statements such as this one by chief warrant Officer Guy Hunter have been forced out of the prisoners. Hunter: I condemn this aggression against peaceful Iraq. Correspondent off: Hunter’s wife says she’s convinced he didn’t mean it.	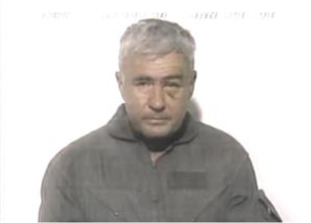
0:41	Hunter: Oh, no, no, no, no, no. He’s/he’s behind – president Bush and his decisions one hundred percent.	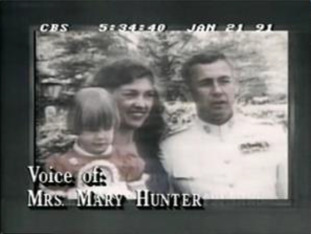
	[40 Sek. über Änderung im code of conduct für Kriegsgefangene]	
1:22	Correspondent off: An Israeli expert on Iraq says that what prisoners may be forced to say	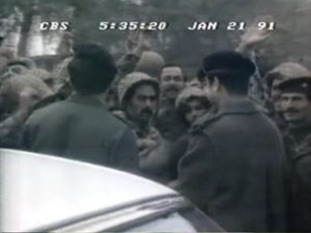
1:26	is less important than sending	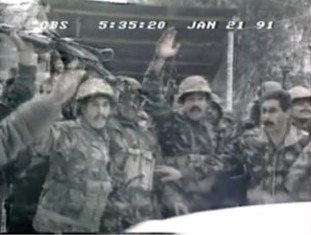
1:28	a strong message back to Saddam and his soldiers.	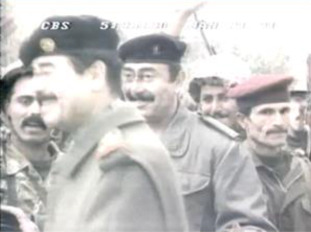
1:31	Baram on {spricht mit Akzent}: If YOU are involved in these atrocities inasmuch as just – touching one of these people with your finger, YOU will be tried as a war criminal and hanged until you die.	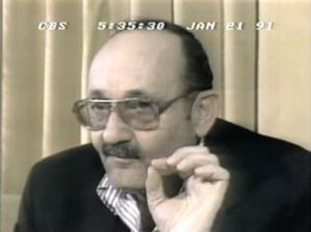
1:43	Correspondent off: In Cherry Hill, New Jersey, Jeffery Zaun’s Navy classmate had a different message.	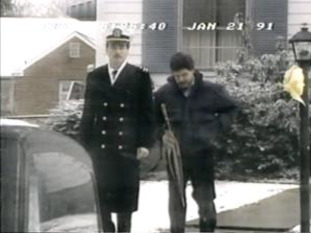
1:50	Braithwaite on: Jeff, we’re praying for ya, and eh/– we’ll do all that we can, of course eh/to take care of your family back here in/eh/in the United States.	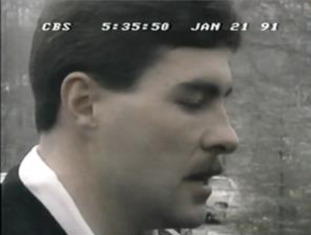
1:57	Correspondent on: But the bottom line, officials know that there ISN’T much that the US can do except to PROTEST the treatment of its prisoners of war. Quoting international LAW to Saddam Hussein – hasn’t stopped him from doing anything YET. – Dan?	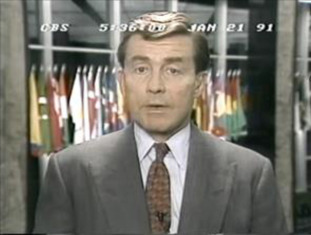
2:11	Anchor off: Thanks, Bill.	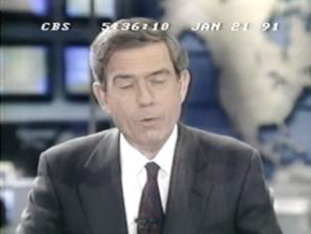

Wir haben es hier mit einem Korrespondentenbericht mit vielen kurzen O‑Tönen zu tun, die in eine übergeordnete News Narrative – die im Kern einer Narration von Gut gegen Böse entspricht – eingebunden sind und argumentativ funktionalisiert werden. Zudem ist in diesem Beitrag eine narrative Themenentfaltung (›erzählen i.e. S.‹) ansatzweise realisiert, weil mindestens vier Zeitebenen involviert sind: Das Gefangenhalten von amerikanischen Soldaten, darauffolgende (z. T. als empörende dargestellte) Reaktionen, der Zeitpunkt der Berichterstattung und ein Blick in die Zukunft. In diesen Zeitebenen spielen nun verschiedene Emotionen eine Rolle, wobei die O‑Töne eine wichtige Rolle spielen – zugleich werden diese auch für verschiedene Argumentationen, die rekonstruiert werden können, funktionalisiert.

So wird in der Anmoderation und in einem ersten Teil des Beitrags die These vertreten, dass die Gefangenen misshandelt worden seien (»mistreatment«, »abuse«). Dies wird gestützt durch die gezeigten Aufnahmen der Gefangenen, u.a. auch solche von einem Gefangenen mit Verletzungen im Gesicht und einer gekrümmten Körperhaltung. Dadurch erscheint der folgende erste O‑Ton – der auf einen einzigen Satz gekürzt ist (»Iraq respects all international conventions«) und keine Erläuterungen enthält – unglaubwürdig, wodurch indirekt über die Bilder und die Anmoderation eine zweite These etabliert wird: Die Iraker lügen. Die postulierte Unaufrichtigkeit der Iraker wird dann gestützt durch den zweiten O‑Ton, der laut Kommentar nicht-diplomatisch ausgefallen sei und so offenbar indirekt zeigen soll, dass die anderen O‑Töne eben unaufrichtig sind. Dies ist allerdings nur eine scheinbare Stützung, denn der zweite O‑Ton bleibt in seiner Bedeutung letztlich vage (»If you want to be safe, – avoid attacking our country«). Durch seine sequenzielle Einbettung in den Beitrag wie auch die vorangehende Bebilderung und den Kommentar des Korrespondenten wird er aber desambiguiert. Auch der folgende O‑Ton eines Gefangenen wird so eingeleitet, dass seine Glaubwürdigkeit infrage gestellt wird (»little doubt that statements such as this one […] have been forced«). Der O‑Ton der Ehefrau wiederum dient dazu, diese These zu stützen, dass die Äusserungen der Gefangenen erzwungen worden sind (»he didn’t mean it«). Gleichzeitig stützt dies dann wiederum die These, dass die Iraker lügen.

In diesem Beispiel – und dies steht in deutlichem Gegensatz zum Beitrag aus den 1960er-Jahren – etabliert der Bericht selbst verschiedene Thesen, die mit den O‑Tönen (mindestens scheinbar) plausibilisiert werden; es werden nicht nur in den O‑Tönen Argumente wiedergegeben. Parallel zu dieser argumentativen Textebene (die Gefangenen werden misshandelt, die Äusserungen der Gefangenen sind erzwungen, die Iraker lügen) weist der Text auch eine Ebene auf, welche darauf angelegt ist, Emotionen zu wecken. Dies hängt teilweise mit der argumentativen Ebene zusammen – so sind die als Lügen dargestellten O‑Töne darauf angelegt, Empörung hervorzurufen, der Experten-O-Ton thematisiert Wiedergutmachung bzw. Rache und derjenige des Freundes Hoffnung. Dieser O‑Ton wird dann allerdings relativiert durch das letzte Wort des Korrespondenten, welcher das Ende der Geschichte offenlässt bzw. ein Szenario von Hilflosigkeit und weiteren erwartbaren Verletzungen des internationalen Rechts entwirft.

Dadurch haben wir – mindestens im ersten Teil des Beitrags – einen narrativen Stil, der nicht mehr »mimetic« ist und die O‑Töne die eigentliche Berichterstattung ausmachen, sondern einen Stil, der O‑Töne und das, worauf sie reagieren, abwechslungsweise und beinahe staccato-artig montiert, kommentiert und z. T. relativiert. Mit Bock (2016, S. 493) können wir hier von einem »diegetic (telling)« Stil reden, in dem die Journalisten »are clearly the ›tellers‹« (Bock 2016, S. 496 f.), wobei in den entsprechenden News Narratives die O‑Töne nur noch kurze, perfekt eingepasste Häppchen sind. Baym (2004, S. 284) spricht deshalb von einer »televisual form, a primarily visual discourse that offers symbolic representations of the world, downplays the voice of the political actor, and celebrates instead the journalists’ work of narration.«

Der entsprechende Bericht der »Tagesschau« ist hingegen im ›mimetischen‹ bzw. ›typografischen‹ Stil gehalten: Nach einer Anmoderation werden die Aufnahmen der Gefangenen gezeigt, welche »vorgeführt« worden seien. Diese Aufnahmen bestehen aus drei auch für die damalige Zeit längeren O‑Tönen (19 bis 28 Sek.), die aneinandergereiht werden. Es folgt ein O‑Ton des damaligen Präsidenten Bush (34 Sek.), der nur kurz eingeleitet wird (»US-Präsident Bush reagiert heftig.«), ebenso der folgende O‑Ton eines irakischen Botschafters (»Iraks Botschafter in Frankreich, Abdul Rezak al-Hashimi«, 23 Sek.). Dieser beendet den Beitrag.

### Gegenwart

2013 sind die O‑Töne von Politiker*innen in der Schweizer »Tagesschau« im Schnitt wieder etwas länger als in der Woche von 1991, sie liegen in der analysierten Woche von 2013 bei 13,7 Sek. Bis heute dürften sich die Dinge nicht wesentlich verändert haben, so liegen auch im folgenden Beitrag vom 22. Juli 2020 die O‑Töne in dieser Grössenordnung (Tab. 4).

**Tab. 4:** Beispiel Schweizer »Tagesschau« vom 22. Juli 2020

Zeit	Text (Transkript)	Film (Transkript)
0:00	Moderatorin on: Wir zeigen aber vorher, wo die amerikanische Regierung unter Donald Trump sonst noch die Muskeln spielen lässt, nämlich gegenüber China. Sie hat Dutzende Diplomaten und Angestellte des chinesischen Konsulates in Houston aufgefordert, die USA zu verlassen, innert 72 Stunden.	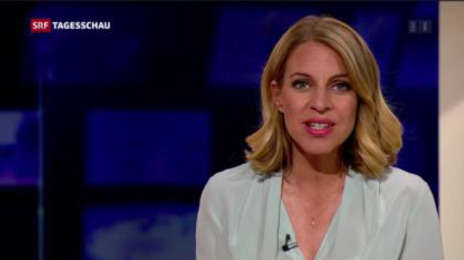
0:18	Korrespondentin off: Mächtig fährt er ein, der amerikanische Aussenminister Mike Pompeo auf seiner gegenwärtigen	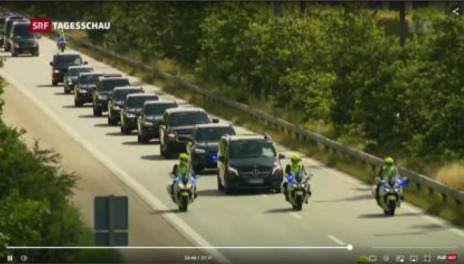
0:24	Europareise. Mit einem gutmütigen Klaps begrüsst er die dänische Premierministerin Mette Frederiksen. Doch nach den Gesprächen	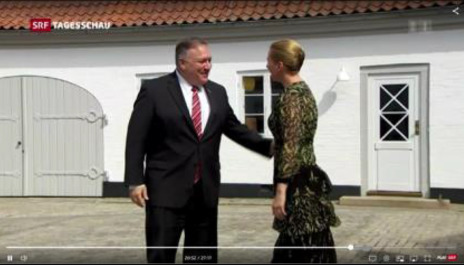
0:32	teilt Pompeo umso heftiger aus. Gegen China.	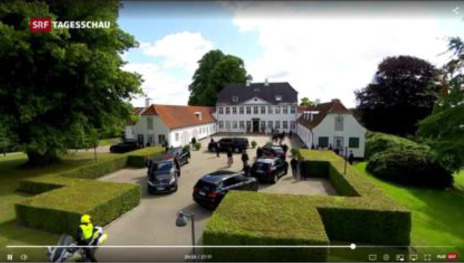
0:35	Pompeo on: There has been this long challenge… Übersetzer off auf Deutsch (im Hintergrund hört man Original-Statement von Pompeo): Seit langem fordert uns die kommunistische Führung Chinas heraus mit dem Diebstahl von geistigem Eigentum. Nicht nur Amerika wurde bestohlen, sondern auch Europa. Das kostete Hundertausende von Jobs in Europa und Amerika. Pompeo on: …all across Europe and America	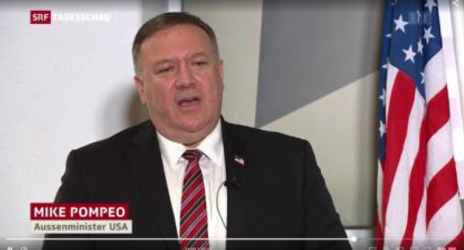
0:50	Korrespondentin off: Warum gerade Chinas Konsulat von Houston	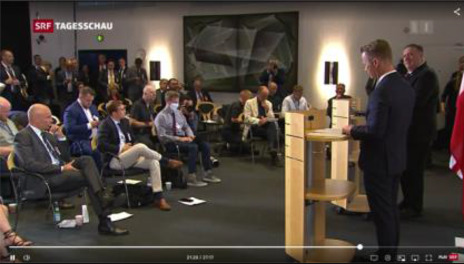
0:54	schliessen muss, sagt Pompeo vor den Medien nicht.	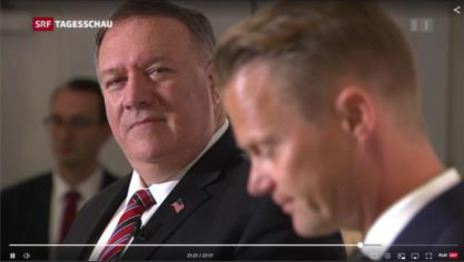
0:56	Feuerwehr und Polizei sind gestern Nacht	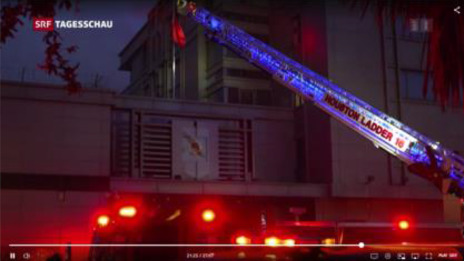
0:59	zum chinesischen Konsulat gerufen worden. Anwohner hatten Feuer auf dem Anwesen	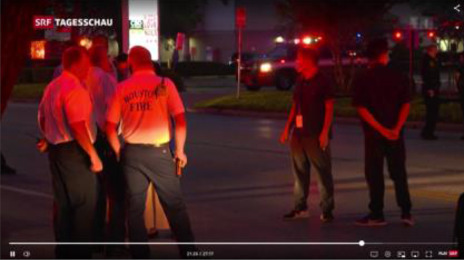
1:03	beobachtet. Lokale Medien berichteten, es würden	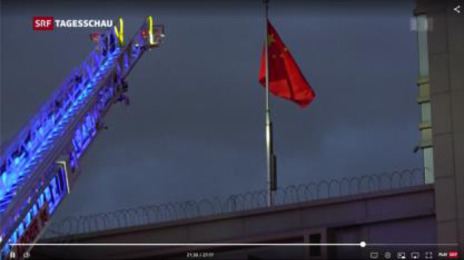
1:06	Dokumente verbrannt. Chinas Aussenministerium dementiert und verurteilt den Schliessungsentscheid vom Dienstag	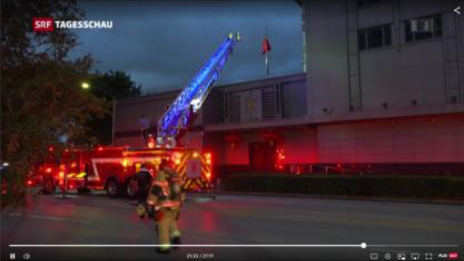
1:12	scharf. Wang Wenbin on: spricht auf Chinesisch Übersetzer off auf Deutsch (im Hintergrund hört man Originalstatement von Wenbin): Dies ist eine politische Provokation der USA und gleichzeitig ein schwerwiegender Verstoss gegen internationales Recht.	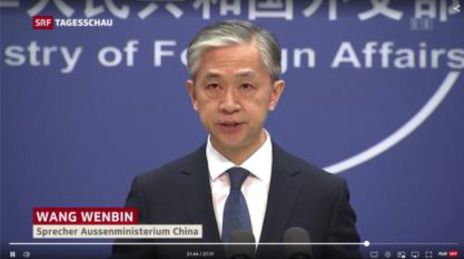
1:23	Korrespondentin off: Die USA müssten auf diesen Entscheid zurückkommen, fordert der Sprecher	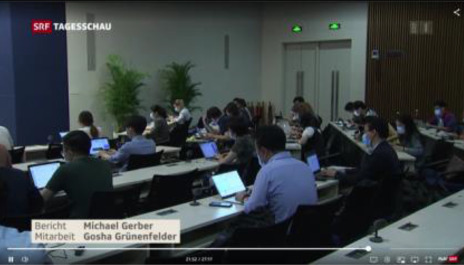
1:26	und droht andernfalls mit Vergeltung.	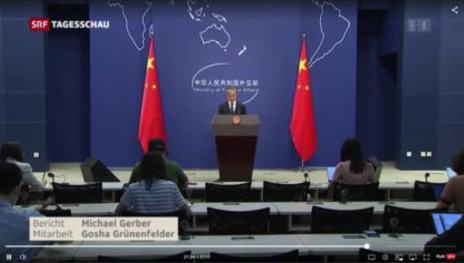
1:29	Moderatorin on: Schon seit Wochen verschlechtert sich das Klima zwischen den USA und China. Nun zieht Trump die Schraube also nochmals an. Peter Düggeli,	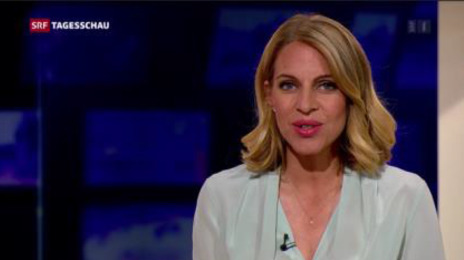
1:37	wie viel ist tatsächliche Bedrohung und wie viel ist schlicht Wahlkampf?	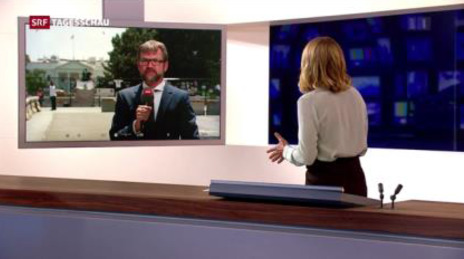
1:43	Korrespondent on: Ja, ich denke, man muss sagen, dass dieser Schritt in dieser Eskalationsstufe schon ein sehr, sehr bedeutender ist. Und ich denke, Präsident Trump sieht das aber alles in allem trotzdem als Wahlkampf. […]	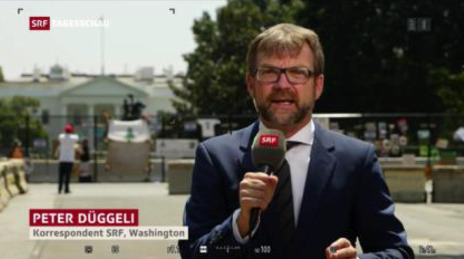

Schon in der Anmoderation wird der ganze Beitrag gerahmt mit der These, dass es beim folgenden Ereignis darum geht, dass Amerika die Muskeln spielen lasse, dass es also um eine Drohgebärde geht. Der ganze Beitrag folgt deutlich der Chronologie der Ereignisse, wobei diese nicht mehr einfach gezeigt werden, sondern deutlich interpretiert (»Mächtig fährt er ein«, »mit einem gutmütigen Klapps«). Diese einführenden Informationen dienen primär der Charakterisierung der ›Hauptfigur‹, die offenbar verschiedene Register ziehen kann (»Doch nach den Gesprächen teilt Pompeo umso heftiger aus«). Es folgt – als dramatischer Höhepunkt in der Mitte des Beitrags – der O‑Ton von Pompeo (17 Sek.), welcher einerseits das schon angekündigte ›Austeilen‹ belegt, aber ebenso die Massnahme der Konsulatsschliessung begründet und so die These der Drohgebärde stützt, weil kein *konkreter* Grund für die Schliessung in Houston genannt wird. Anschliessend wird – nun wieder eher ›mimetisch‹ – die darauffolgende Reaktion des chinesischen Aussenministers ausschnittweise gezeigt (O-Ton 7 Sek.). Die Moderatorin resümiert interpretierend (»nun zieht Trump die Schraube nochmals an«) und leitet dann zu einer Frage an den Korrespondenten vor Ort über. Dieser deutet das Ereignis als eines, das vor allem im Kontext des damals laufenden Wahlkampfs in den USA zu verstehen sei und damit eher als symbolischer Akt.

Der Vergleich mit dem entsprechenden Beitrag der »CBS Evening News« zeigt, dass die amerikanische Sendung hier noch deutlicher interpretiert und deutlich ›diegetischer‹ berichtet als die Schweizer »Tagesschau« (Tab. 5).

**Tab. 5:** Beitrag der »CBS Evening News«

Zeit	Text (Transkript)	Film (Transkript)
0:00	Anchor on: Tonight China is vowing to retaliate after the US accused the country of hacking into companies doing corona virus vaccine research. An order [that’s?] consulate in Houston to close. Now, this is a significant escalation in a growing conflict between the world’s two largest economies, all made worse by the pandemic. Here is CBSes Margaret Brennan.	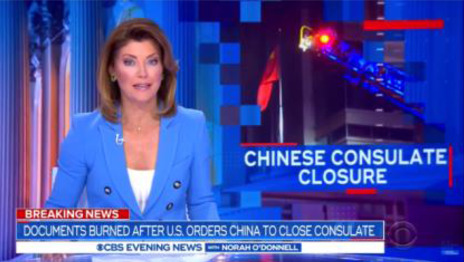
0:23	Correspondent off: Plumes of smoke	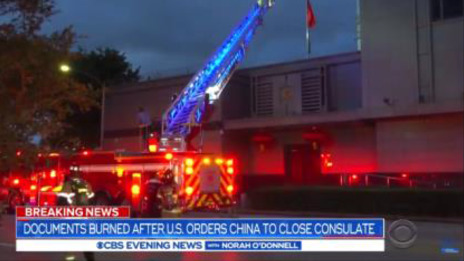
0:24	tipped off firefighters that	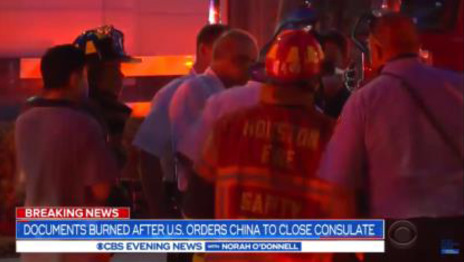
0:26	something was a [mess?] at China’s Houston consulate.	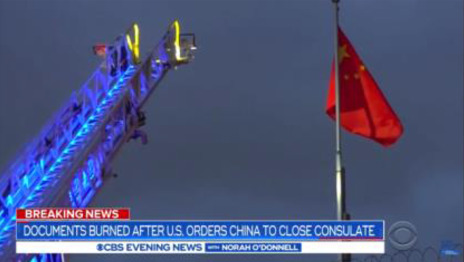
0:29	Firefighter/Policeman on: When I [unverständlich] in to make entry they were denied access to the facility.	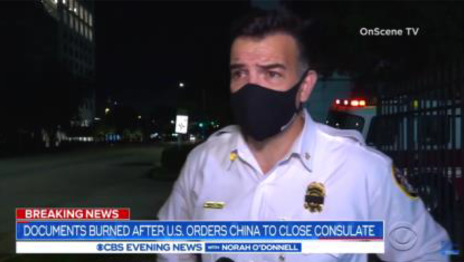
0:32	Correspondent off: These images which have not been verified show people burning documents, a common practice when a diplomatic post is quickly abandoned	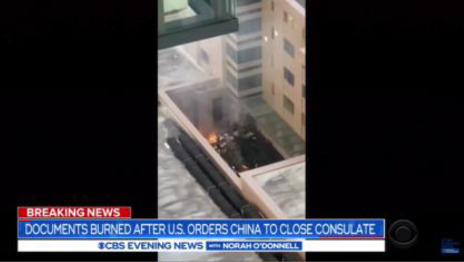
0:40	US officials claim that Houston was a hub for espionage.	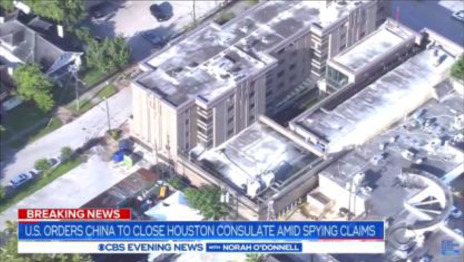
0:44	And that China recently escalated its theft of intellectual property from US institutions.	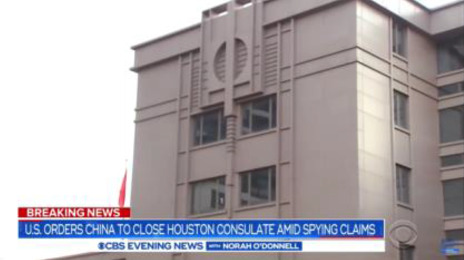
0:49	Hours earlier the state department ordered China’s ambassador to shut down the Texas outpost by Friday.	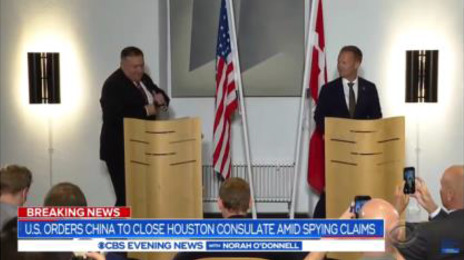
0:55	Secretary of state Mike Pompeo (man hört Mike Pompeo im Hintergrund sprechen). Mike Pompeo on: We are setting out clear expectations for how the Chinese communist party is going to behave. And when they don’t, we gonna take actions to protect the American people, protect our security, our national security and also protect our economy and jobs.	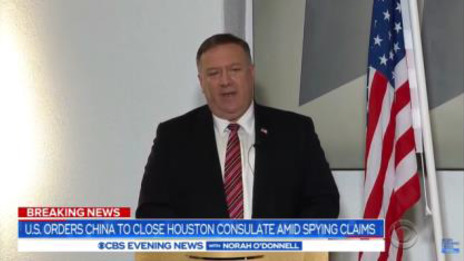
1:10	(Man hört Wenbin im Hintergrund auf Chinesisch sprechen) An outraged Beijing threatened there may be consequences for expelling dozens of its diplomats.	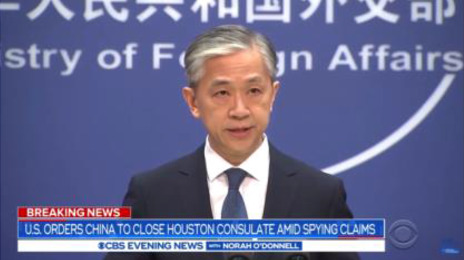
1:18	Tensions with Beijing have escalated since the pandemic began.	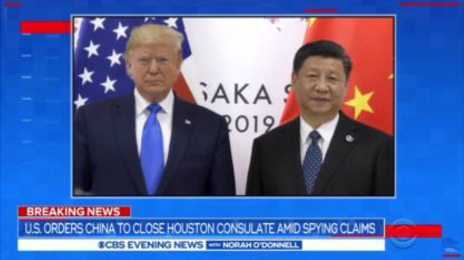
1:21	Trump on: Kung-Flu	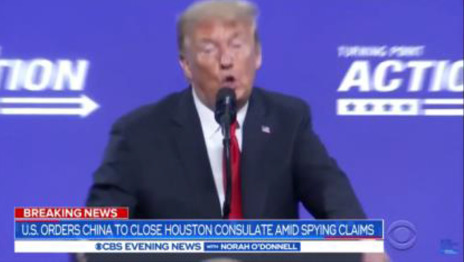
1:22	Trump on: It’s a nasty, horrible disease that should have never been allowed to escape China.	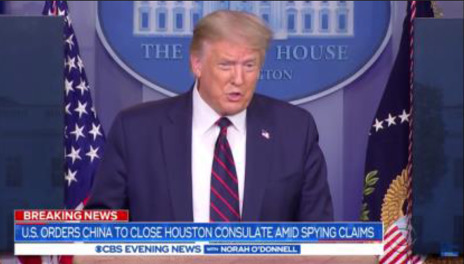
1:28	Correspondent off: And President Trump’s long promised trade deal has stalled.	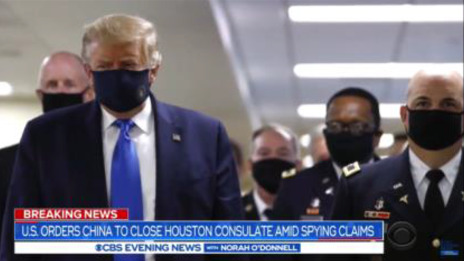
1:31	Trump on: I’m not interested right now in talking to China.	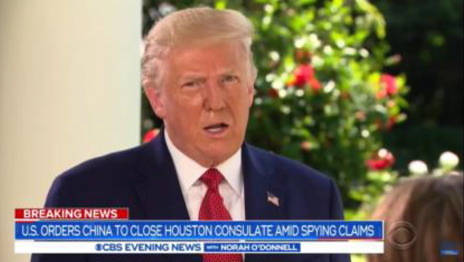
1:33	Correspondent off: China claims it is all unfair stigmatizing for political reasons.	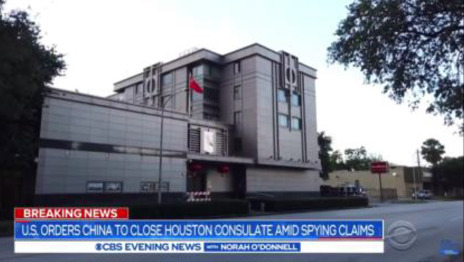
1:39	Correspondent on: Now sources tell CBS news that China’s espionage has gone increasingly bracing at medical facilities and universities Accredited Chinese diplomats have even gotten caught trying to sneak into military bases in Florida and Virginia and at Houston’s airport carrying fake IDs	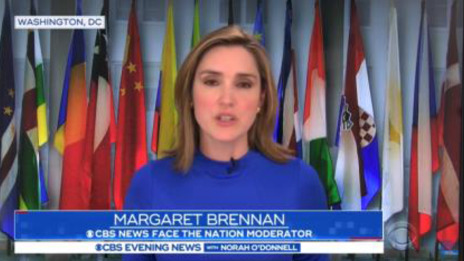
2:00	trying to help Chinese nationals board a chartered plane, Norah. Anchor on: Pretty bracing. Margaret Brennan, thank you.	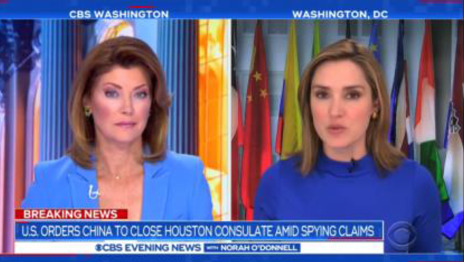

Dieser Beitrag vertritt die These, dass den Chinesen nicht zu trauen ist, sie etwas zu verbergen haben und dass sie spionieren – was wiederum die Anklage der USA indirekt stützt. So kommt ganz zu Beginn ein Feuerwehrmann zu Wort, der berichtet, der Zugang zum Gebäude sei ihnen verwehrt worden, es werden (nicht verifizierte) Bilder von Personen, die Dokumente verbrennen, gezeigt – um gleichzeitig die Behauptung zu wiederholen, das Konsulat sei ein Hort für Spionage gewesen. Dann wird in einer Art Rückblende – und durch diese sequenzielle Anordnung erscheint das Vernichten der Papiere als direkte Reaktion darauf – der O‑Ton von Pompeo gezeigt, welcher die (vermeintliche) Reaktion der Chinesen als die Folge einer erfolgreichen Drohung erscheinen lässt – und so noch einmal die These stützt, dass die Chinesen etwas zu verbergen haben. Dass die chinesische Regierung dies dementiert, wird nicht berichtet, sondern lediglich, dass sie erzürnt sei.

Dann folgt ein Rückblick auf Präsident Trumps Verhalten, welcher den Konflikt in einen grösseren Kontext der Corona-Pandemie stellt. Dieser Rückblick besteht im Kern aus drei sehr kurzen (1 Sek., 6 Sek., 3 Sek.) O‑Tönen, in denen Trump sich zu China äussert – wobei hier staccato-artig ein Äusserungsfragment und zwei einzelne Sätze zusammengeschnitten worden sind. Dabei ist insbesondere der Übergang zwischen dem ersten und zweiten O‑Ton äusserst auffällig gestaltet, als die Filmaufnahme des ersten O‑Tones gross gezoomt wird und von der Mitte her die Aufnahme des zweiten O‑Tones erscheint. Diese Sequenz ist ein Beispiel dafür, wie heutige Fernsehnachrichtenbeiträge komplexe narrative Verfahren (hier einen Rückblick) verwenden, die sehr deutlich als gestaltete Elemente erkennbar sind und gerade auch durch ihre Form auf Unterhaltung angelegt sind. Gleichzeitig wird hier die Illusion der unvermittelten Wirklichkeitsabbildung gebrochen. Im Sign-off führt die Korrespondentin dann weitere Informationen an, welche die These stützen, dass China nicht zu trauen ist; die Moderatorin, die auch im Bild ist, schüttelt dazu leicht den Kopf und zeigt so ihre Empörung an.

## Fazit

Die Berichterstattung in Fernsehnachrichten re-konstruiert Wirklichkeit; dies muss immer selektiv und damit interpretativ erfolgen. Diesem Umstand wird ein Verständnis von Fernsehnachrichtenbeiträgen als ›News Narrative‹ gerecht, welches zudem ermöglicht einzelne gestalterische Mittel – verbale, filmische, akustische – in einem theoretischen Rahmen und in ihrem gestalthaften Zusammenhang zu sehen.

Die Analyse der O‑Töne von Politiker*innen und Behördenvertreter*innen in der politischen Berichterstattung der Schweizer »Tagesschau« und der amerikanischen »CBS Evening News« hat gezeigt, dass es vergleichbare Trends, aber auch Unterschiede zwischen den Sendungen gibt. So bewegen sich in der »CBS Evening News« die O‑Töne aller Akteur*innen seit den frühen 1980er-Jahren um eine durchschnittliche Länge von 10 Sek. In der Schweizer »Tagesschau« hingegen setzt ein Trend zu kürzeren O‑Tönen erst in den frühen 1990er-Jahren ein und im Schnitt bleiben diese länger. Es kann also grundsätzlich von einem identischen Trend gesprochen werden, der in den USA aber früher einsetzt und ausgeprägter beobachtet werden kann.

Schaut man sich diese Entwicklung mit einem Fokus auf Narration und die argumentative Funktionalisierung von O‑Tönen an, dann kann ebenfalls in beiden Sendungen derselbe Trend beobachtet werden: weg vom ›mimetischen‹ Berichten, in dem tendenziell so getan wird, als würde eine aussermediale Realität unverändert abgebildet und als würden die O‑Töne selbst das Rückgrat der Informationen ausmachen, hin zu einem ›diegetischen‹ Stil, welcher den Akt des Erzählens deutlicher zeigt, in dem aber auch die journalistische Interpretation im Zentrum steht und die O‑Töne narrativ zugeschnitten werden und so ereignisbezogene Thesen gezielt stützen. Auch hier zeigt sich, dass dieser Trend in der »CBS Evening News« (deutlich) früher und ausgeprägter zu beobachten ist. Mit diesem Wandel vom ›mimetischen‹ Berichten hin zum ›diegetischen‹ Erzählen einher geht eine Art Verlagerung der Argumentation: Während in den älteren Berichten aus den 1960er-Jahren Argumentationen überwiegend in den O‑Tönen selbst realisiert werden und Argumentation so in den Berichten abgebildet bzw. eben ›berichtet‹ wird, so liegt die argumentative Hoheit in den Berichten etwa ab den 1990er-Jahren zunehmend auf Seiten der Journalist*innen. Die eigentlichen Thesen werden – oft implizit und damit nicht gut einklagbar – in den journalistischen Textpassagen aufgestellt, die kürzer werdenden O‑Töne dienen der Plausibilisierung dieser journalistischen Thesen. Dabei ermöglicht es die Kürzung der O‑Töne, die daraus entstehenden Häppchen flexibel und gezielt in die (auch argumentativ abgestützte) Wirklichkeitsdarstellung der Journalist*innen einzubetten. Insgesamt werden die Berichte damit in dem Sinne narrativer, als ein ganzes Ereignis übergreifende Thesen vertreten werden, welche dann durch eine entsprechende Kombination von journalistischem Text und O‑Tönen plausibilisiert werden, wobei die O‑Töne selbst die Thesen meist nur auf den ersten Blick wirklich eindeutig stützen. Zudem können auch in der zunehmenden Emotionalisierung und in chronologischen Themenentfaltungen weitere Elemente einer ›Narrativisierung‹ der Berichterstattung gesehen werden.

Bezogen auf journalistische Kulturen (vgl. Hanitzsch 2007; Luginbühl 2014) können diese Trends einerseits auf eine zunehmende Marktorientierung der Sendungen bezogen werden, wobei diese in der Schweizer »Tagesschau« offenbar später einsetzt. Zudem kann dieser Trend bezogen werden auf einen Wandel in Bezug auf den ›Objektivismus‹ (vgl. Hanitzsch 2007) der Berichterstattung; dieser verändert sich dahingehend, dass die Konstruktionsleistung zunehmend weniger versteckt oder sogar offen gezeigt oder thematisiert wird. Gleichzeitig verändert sich damit der ›Empirismus‹ (vgl. Hanitzsch 2007); es wird weniger so getan, als würden die Fakten (oder im vorliegenden Fall die O‑Töne) für sich selbst sprechen, sondern es steht die Deutung eines Ereignisses im Zentrum, welche argumentativ-analytisch plausibilisiert wird. Dabei haben wir es aber in den allermeisten Fällen nicht mit explizit markierten Argumentationen zu tun, sondern mit impliziten.

Die Narration und damit verbunden der gezielte narrative Zuschnitt von O‑Tönen wird offensichtlicher und tendenziell wird so der Anspruch auf unhinterfragbare Objektivität früherer Berichterstattung relativiert. Damit verbunden ist ein journalistisches Risiko, dass diese Art der offensichtlich narrativen Aufbereitung die Vertrauenswürdigkeit unterwandert und die journalistische Autorität unterminiert. Vielleicht ist es dieses Risiko, das minimiert werden soll, indem Argumentationen und die argumentative Funktionalisierung von O‑Tönen in aller Regel implizit bleiben; damit bleiben auch die entsprechenden Interpretationen implizit.

## Footnotes

1 S. dazu für die Schweiz etwa https://www.persoenlich.com/medien/die-fernsehsender-sind-im-quotenhoch, für Deutschland https://meedia.de/2020/03/17/tv-konsum-in-corona-zeiten-tagesschau-und-heute-gewinnen-den-tag-hart-aber-fair-mit-bester-zahl-seit-2016-merkel-pk-holt-bei-ntv-107/, für die USA https://www.nytimes.com/2020/03/24/business/media/coronavirus-evening-news.html.

2 Ich verstehe hier und im Folgenden unter »Text« multimodale Kommunikate und nicht nur deren verbalen Anteil.

3 Was ich hier und im Folgenden nicht berücksichtige, sind Interviews in eigenständigen Beiträgen oder im Nachrichtenstudio (dazu Fetzer/Bull 2013).

4 Zum Korpus s. Kapitel 4 und Luginbühl (2014, S. 137–149).

5 Im Vergleich zu anderen Personengruppen (Behördenvertreter*innen, Expert*innen, Privatpersonen, Militärangehörige, Andere) lässt sich keine einheitliche Entwicklung beobachten.

6 Ich verstehe unter Filmmeldungen die Kombination von Nachrichtenfilm und einer anonymen Stimme aus dem Off, unter Korrespondentenberichten längere Beiträge, in denen die Autorin/der Autor eines Beitrags namentlich genannt wird und oft (aber nicht immer) im Bild zu sehen ist. Zur genaueren Bestimmung s. Luginbühl (2014, S. 121, 127).

7 152 Beiträge mit O‑Tönen von Politiker*innen (CBS Evening News: 72 Beiträge, davon 16 auch mit Behördenvertreter*innen; Tagesschau: 80 Beiträge, davon 6 auch mit Behördenvertreter*innen); nur mit Behördenvertreter*innen 32 Beiträge bei CBS, 17 bei Tagesschau.

8 Die Beiträge der »Tagesschau« wurden bis in die 1980er-Jahre ohne den live gesprochenen Sprechertext archiviert. Im Transkript wird das archivierte Sprechermanuskript wiedergegeben. Aufgrund einiger erhaltener Tondokumente ist davon auszugehen, dass der Text eher langsam und monoton verlesen worden ist.

## References

[CR1] Baym, Geoffrey (2004): Packaging Reality: Structures of Form in US Network News Coverage of Watergate and the Clinton Impeachment. In: *Journalism* 5, S. 279–299.

[CR2] Bird, Elizabeth S./Dardenne, Robert W. (1988): Myth, Chronicle, and Story. Exploring the Narrative Qualities of News. In: James W. Carey (Hg.): *Media, Myths, and Narratives: Television and the Press*. Beverly Hills, CA: Sage (Sage annual reviews of communication research 15), S. 67–86.

[CR3] Bird, Elizabeth S./Dardenne, Robert W. (2009): Rethinking news as myth and storytelling. In Karin Wahl-Jorgensen/Thomas Hanitzsch (Hg.): *Handbook of Journalism Studies*. New York: Routledge, S. 205–217.

[CR4] Bock, Mary Angela (2016): Showing versus telling: Comparing online video from newspaper and television websites. In: *Journalism* 17, S. 493–510.

[CR5] Bucy, Erik P./Grabe, Maria Elizabeth (2007): Taking Television Seriously: A Sound and Image Bite Analysis of Presidential Campaign Coverage, 1992–2004. In: *Journal of Communication* 57, S. 652–675.

[CR6] Buozis, Michael/Creech, Brian (2018): Reading News as Narrative. In: *Journalism Studies *19, S. 1430–1446.

[CR7] Dunn, Anne (2005): Television news as narrative. In: Anne Dunn/Helen Fulton/Julian Murphet/Rosemary Huisman (Hg.): *Narrative and Media*. Cambridge: Cambridge University Press, S. 140–152.

[CR8] Epstein, Edward Jay (1974): *News from nowhere: Television and the news*. New York: Random House.

[CR9] Esser, Frank (2008): Dimensions of Political News Cultures: Sound Bite and Image Bite News in France, Germany, Great Britain and the United States. In: *International Journal of Press/Politics* 13, S. 401–428.

[CR10] Fetzer, Anita/Bull, Peter (2013): Political interviews in context. In: Piotr Cap/Urszula Okulska (Hg.): *Analyzing Genres in Political Communication. Theory and practice*. Amsterdam: Benjamins, S. 73–100.

[CR11] Genette, Gérard (1998): *Die Erzählung*. 2. Auflage. München: UVK.

[CR12] Grundler, Elke (2011): *Kompetent argumentieren. Ein gesprächsanalytisch fundiertes Modell*. Tübingen: Stauffenburg.

[CR13] Hallin, Daniel C. (1992): Sound bite news: Television coverage of elections, 1968–1988. In: *Journal of Communication* 42, S. 5–24.

[CR14] Hanitzsch, Thomas (2007): Journalismuskultur: Zur Dimensionierung eines zentralen Konstrukts der kulturvergleichenden Journalismusforschung. In: *Medien & Kommunikationswissenschaft *55, S. 372–389.

[CR15] Johnson-Cartee, Karen S. (2005): *News narratives and news framing. Constructing political reality*. Lanham: Rowman & Littlefield.

[CR16] Kienpointner, Manfred (2008): Argumentationstheorie. In: Ulla Fix/Andreas Gardt/Joachim Knape (Hg.): *Rhetoric and Stylistics / Rhetorik und Stilistik, Teilband 1*. Berlin, New York: Mouron de Gruyter, S. 702–717.

[CR17] Krieken, Kobie van (2016): *Linguistic Viewpoint in Crime News Narratives*. Utrecht: LOT.

[CR18] Lichter, S. Robert (2001): A Plague on Both Parties: Substance and Fairness in TV Election News. In: *Harvard International Journal of Press/Politics* 6, S. 8–30.

[CR19] Linke, Angelika (2009): Stil und Kultur. Ulla Fix/Andreas Gardt/Joachim Knape (Hg.): *Rhetoric and Stylistics / Rhetorik und Stilistik, Teilband 2*. Berlin, New York: Mouton de Gruyter, S. 1131–1144.

[CR20] Lippmann, Walter (1921): *Public Opinion*. New York: Rütten & Loening.

[CR21] Lowry, Dennis T./Shidler, Jon A. (1995): The sound bites, the biters, and the bitten: An analysis of network TV news bias in campaign ’92. In: *Journalism and Mass Communication Quarterly* 72, S. 33–45.

[CR22] Lowry, Dennis T./Shidler, Jon A. (1998): The sound bites, the biters, and the bitten: A two-campaign test of the anti-incumbent bias hypothesis in network TV news. In: *Journalism and Mass Communication Quarterly* 75, S. 719–729.

[CR23] Luginbühl, Martin (2004): Staged authenticity in TV news. In: *Studies in Communications Sciences* 4, S. 129–146.

[CR24] Luginbühl, Martin (2014): *Medienkultur und Medienlinguistik. Komparative Textsortengeschichte(n) der amerikanischen »CBS Evening News« und der Schweizer »Tagesschau«*. Bern: Lang.

[CR25] Luginbühl, Martin/Schwab, Kathrine/Burger, Harald (2004): *Geschichten über Fremde. Eine linguistische Narrationsanalyse von Schweizer Fernsehnachrichten von 1957 bis 1999*. Bern: Lang.

[CR26] Maurer, Torsten/Wagner, Matthias/Weiß, Hans-Jürgen (2020): Fernsehnachrichten: Mehr als Klimawandel, Brexit, Europa- und Landtagswahlen. In: *media perspektiven* 2, S. 62–86.

[CR27] Montgomery, Martin (2007): *The discourse of broadcast news. A linguistic approach*. London: Routledge.

[CR28] Neiger, Motti/Tenenboim-Weinblatt, Keren (2016): Understanding Journalism Through a Nuanced Deconstruction of Temporal Layers in News Narratives. In: *Journal of Communication* 66, S. 139–160.

[CR29] Roeh, Itzhak (1989): Journalism as Storytelling, Coverage as Narrative. In: *American Behavioral Scientist* 33, S. 162–168.

[CR30] Russomanno, Joseph A./Everett, Stephen E. (1995): Candidate Sound Bites: Too Much Concern Over Length? In: *Journal of Broadcasting & Electronic Media* 39, S. 408–415.

[CR31] Schudson, Michael (1982): The Politics of Narrative Form: The Emergence of News Conventions in Print and Television. In: *Daedalus* 111, S. 97–112.

[CR32] Shim, Hoon (2014): Narrative journalism in the contemporary newsroom: The rise of new paradigm in news format? In: *Narrative Inquiry *24, S. 77–95.

[CR33] Sülflow, Michael/Esser, Frank (2014): Visuelle Kandidatendarstellung in Wahlkampfbeiträgen deutscher und amerikanischer Fernsehsender – Image Bites, Rollenbilder und nonverbales Verhalten. In: *Publizistik* 59, S. 285–306.

[CR34] Tenenboim Weinblatt, Keren (2008): Fighting for the story’s life: Non-closure in journalistic narrative. In: *Journalism* 9, S. 31–51.

[CR35] Toulmin, Stephen E. (2003 [1958]): *The Uses of Argument. Updated Edition*. Cambridge: Cambridge University Press.

[CR36] Zelizer, Barbie (1990): Achieving journalistic authority through narrative. *Critical Studies in Mass Communication *7, 366–376.

